# Characterization of Supersonic Compressible Fluid Flow Using High-Speed Interferometry

**DOI:** 10.3390/s21238158

**Published:** 2021-12-06

**Authors:** Pavel Psota, Gramoz Çubreli, Jindřich Hála, David Šimurda, Petr Šidlof, Jan Kredba, Marek Stašík, Vít Lédl, Michal Jiránek, Martin Luxa, Jan Lepicovsky

**Affiliations:** 1 Faculty of Mechatronics, Informatics and Interdisciplinary Studies, Technical University of Liberec, Studentská 2, 461 17 Liberec, Czech Republic; pavel.psota@tul.cz (P.P.); Petr.Sidlof@tul.cz (P.Š.); jan.kredba@tul.cz (J.K.); marek.stasik@tul.cz (M.S.); vit.ledl@tul.cz (V.L.); michal.jiranek@tul.cz (M.J.); 2 Faculty of Mechanical Engineering, Technical University of Liberec, Studentská 2, 461 17 Liberec, Czech Republic; 3 Institute of Thermomechanics of the Czech Academy of Sciences, Dolejškova 1402/5, 182 00 Praha, Czech Republic; hala@it.cas.cz (J.H.); simurda@it.cas.cz (D.Š.); luxa@it.cas.cz (M.L.); jandrsc@gmail.com (J.L.)

**Keywords:** high-speed, interferometry, supersonic, compressible flow, wind tunnel

## Abstract

This paper presents a very effective interference technique for the sensing and researching of compressible fluid flow in a wind tunnel facility. The developed technique is very sensitive and accurate, yet easy to use under conditions typical for aerodynamic labs, and will be used for the nonintrusive investigation of flutter in blade cascades. The interferometer employs a high-speed camera, fiber optics, and available “of-the-shelf” optics and optomechanics. The construction of the interferometer together with the fiber optics ensures the high compactness and portability of the system. Moreover, single-shot quantitative data processing based on introducing a spatial carrier frequency and Fourier analysis allows for almost real-time quantitative processing. As a validation case, the interferometric system was successfully applied in the research of supersonic compressible fluid discharge from a narrow channel in a wind tunnel. Density distributions were quantitatively analyzed with the spatial resolution of about 50 μm. The results of the measurement revealed important features of the flow pattern. Moreover, the measurement results were compared with Computational Fluid Dynamics (CFD) simulations with a good agreement.

## 1. Introduction

High-speed, high-accuracy, whole-field, non-destructive and contactless optical methods are increasingly being used in the study of fluid flow, offering the possibility for deeper insights than single-point conventional techniques such as constant-current anemometry [1], pneumatic measurements [2], thermal flow sensors (hot film sensors, calorimetric sensors, time-of-flight sensors) [3,4,5,6] and others. Moreover, contactless optical methods do not disturb the flow field. Due to the presence of density gradients, compressible fluid flow is very suitable for an investigation using optical methods that are sensitive either to the refractive index of the medium through which the light wave propagates (interferometry), or to a refractive index gradient in the medium (schlieren [7,8,9,10,11], shadowgraph [12,13,14,15,16], Moiré deflectometry [17,18,19,20,21]). Interferometric methods such as classical interferometry [22,23], holographic interferometry [24] and digital holographic interferometry [25,26,27,28,29] stand as the most accurate optical techniques [26,30,31] that are applied in many sectors of industry and scientific research, notably in the study of liquid cooling [32], diffusion [33], convection [34,35], temperature measurements and visualization [1,25,36], temperature fields measurement in pulsatile jets [25], studies of living cell imaging [37,38], precision measurement [15,39,40], vibrometry [41,42] etc.

However, the field of the transonic or even supersonic flow generated in wind tunnel facilities is very specific, and the current state of the art of interferometric techniques in this field allows only their limited use. Environmental harshness, particularly vibrations, the lack of an optical table, temporal flow instability, and high fluid density gradients leading to complex interference patterns often make the quantitative evaluation of interferometric data very difficult and cumbersome. This is why the use of brute force interferogram analysis [43] or capturing interferograms for qualitative analysis and flow visualization in wind tunnels is still common [22,44,45,46,47,48,49].

In this paper, we introduce a robust, portable, high-speed and high-resolution interferometric method that can be easily applied in a wind tunnel facility, providing very accurate results with high spatial resolution in almost real time. The method utilizes a high-speed camera allowing for short exposures to avoid the blur of interference patterns typical for unstable flow, as well as to minimize environmental disturbances. Fiber optics ensure the high compactness and portability of the interferometric system, and fast single-shot quantitative data processing based on spatial carrier frequency and Fourier analysis is employed.

The method is intended for the investigation of high-speed flow in planar blade cascades, namely, for studying dynamic events during transonic and supersonic blade flutter. As a first step, it was successfully tested in the case of a supersonic highly underexpanded flow at the outlet of a narrow channel by measuring the density distribution of the flow. The present interferometric technique has proven to be an excellent tool to meet these objectives. In addition to a description of the principles, experimental arrangement and results, numerical simulations showing the high reliability of the measured data are presented.

## 2. Method

### 2.1. Interferometry and Fluid Flow

Interferometric techniques generally provide whole-field, non-invasive and highly accurate measurements. These properties can be advantageously used for measuring and visualizing such flow phenomena that affect light wave [25]. The amplitude of the propagating light wave through the transparent/semi-transparent medium under investigation is not significantly affected, while the optical phase is [50]. These phenomena are called phase-sensitive, implying that when a change takes place in the medium, it affects the refractive index of the medium Δn, which as a consequence introduces phase deviation Δφ:

(1) $$\Delta \varphi \left(x,y\right)=\frac{2\pi }{\lambda }\int_{L_{1}}^{L_{2}}\Delta n\left(x,y,z\right)dl,$$

with dl denoting the differential distance along the optical path between points $L_{1}$ and $L_{2}$, and λ stands for the wavelength of the laser light. The x,y coordinates define the position within the Cartesian coordinates system. The general solution of (1) leads to tomographic techniques [51] that are difficult or cumbersome to apply in real-time, dynamic measurements. However, there are two assumptions that allow a significant simplification of (1): (i) wind tunnel experiments are designed in order to keep the refractive index along the z-axis constant, i.e., the optical axis, and (ii) the bending of the rays due to a spatial variation in the refractive index can be neglected. Assuming (i) and (ii), Equation (1) can be simplified to:

(2) $$\Delta \varphi \left(x,y\right)=\frac{2\pi }{\lambda }\Delta n\left(x,y\right)\;L,$$

where L denotes the length of the object in the z-axis direction (i.e., wind tunnel testing section width). The refractive index change distribution is usually not the main concern. An important equation in the optical study of fluid flow is the Gladstone–Dale equation, which links the change of refractive index Δn to the density ρ as:

(3) $$\rho \left(x,y\right)=\rho_{ref}+\frac{\Delta n\left(x,y\right)}{K},$$

where K represents the Gladstone–Dale constant and $\rho_{ref}$ is the reference density. The Gladstone–Dale equation is almost independent of pressure or temperature under moderate physical conditions [50]. For a case of an isentropic compressible flow of ideal gas, the measured outlet density ρ can be further linked with the isentropic Mach number by the following equation:

(4) $$M_{i}\left(x,y\right)=\sqrt{\frac{2}{\kappa -1}\cdot \left({\left(\frac{\rho_{in}}{\rho \left(x,y\right)}\right)}^{\kappa -1}-1\right)},$$

where $\rho_{in}$ is the inlet air density and κ=1.4 denotes the heat capacity ratio of an ideal diatomic gas. In addition, other quantities, such as velocity distribution, pressure distribution, temperature distribution, etc., can be analyzed quantitatively by interferometry.

**Figure 1:**
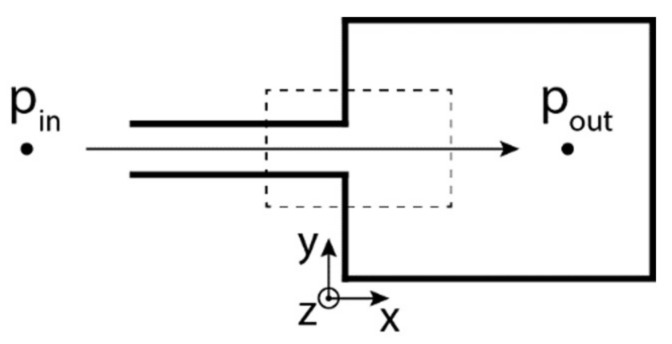
Basic concept of the narrow channel measurement. Dashed rectangle represents the investigated area.

The investigation within this paper leads to the characterization of the air flow in/behind a narrow channel (see Figure 1). Air enters the test section at ambient conditions, and thus the total pressure $p_{in}$ is equal to the barometric pressure. The static pressure at the outlet of the narrow channel $p_{out}$ is measured by a pressure tap on a wall in the settling chamber using a pressure transducer (from Huba Control AG, Würenlos, Switzerland). For the case of an isentropic compressible flow of ideal gas, the density distribution $\rho_{M}$ in the settling chamber can be calculated as:

(5) $$\rho_{M}\left(x,y\right)=\rho_{ref}+\frac{\Delta n\left(x,y\right)}{K},$$

where

(6) $$\rho_{ref}=\frac{p_{out}}{RT_{0}}.$$

In (6), $T_{0}$ denotes the ambient air temperature and $R\;=\;287.1\;J/\left(\mathrm{kg}\cdot K\right)$ is the specific gas constant. The isentropic Mach number (4), however, can provide relevant information only in flows where the drops in total pressure and total density throughout the flow field are relatively small, e.g., the flow in blade cascades. This does not apply to the investigated phenomenon, and therefore the isentropic Mach number was not evaluated.

### 2.2. Spatial Carrier Interferometry

Equations (2)–(6) provide the relation between the phase change Δφ and other quantities, such as density $\rho_{M}$; however, it is not clear yet how the phase change Δφ is obtained.

The interference phase φ can be retrieved from an interference pattern

(7) $$I\left(x,y\right)=A\left(x,y\right)+B\left(x,y\right)\;\cos\left(\varphi \left(x,y\right)\right)$$

created on a charge-coupled device (CCD), or a complementary metal-oxide semiconductor (CMOS) chip, by the superposition of a reference and an object beam. In (7), A is the additive component and B is the multiplicative component. The interference phase φ is coded in a cosine modulate fringe pattern. There are several ways to retrieve the interference phase [52]; however, for the characterization of fluid flow in wind tunnels, where real-time and high-resolution measurements are required, most of the interferogram processing techniques fail. An option to tackle the issues is spatial carrier interferometry [52], which introduces small angles of incidence $\theta_{x}$, $\theta_{y}$ in the x, y directions on the camera sensor in the reference beam. This results in further modulation of the interference pattern with cosine fringes pattern of constant spatial frequencies $k_{x0}=\frac{2\pi }{\lambda }sin\theta_{x}$ and $k_{y0}=\frac{2\pi }{\lambda }sin\theta_{y}$, respectively. Let us rewrite (7) using a complex exponential including the carrier frequencies $k_{x0}$ and $k_{y0}$:

(8) $$I\left(x,y\right)=A\left(x,y\right)+1/2C\left(x,y\right)+1/2C^{*}\left(x,y\right),$$

where $C\left(x,y\right)=B\left(x,y\right)\exp\left[i\left(\varphi \left(x,y\right)+k_{x0}x+k_{y0}y\right)\right]$ and $C^{*}\left(x,y\right)=B\left(x,y\right)\exp\left[-i\left(\varphi \left(x,y\right)+k_{x0}x+k_{y0}y\right)\right]$, with imaginary unit i. Equation (8) in the Fourier domain

(9) $$\hat{I}\left(k_{x},k_{y}\right)=\hat{A}\left(k_{x},k_{y}\right)+\hat{C}\left(k_{x}-k_{x0},k_{y}-k_{y0}\right)+{\hat{C}}^{*}\left(k_{x}+k_{x0},k_{y}+k_{y0}\right)$$

with the spatial frequency coordinates $k_{x},\;k_{y}$ is composed of a central DC (direct current) term and two conjugated components located symmetrically from the center of the spectrum. The roof symbol ^ denotes Fourier spectrum and the superscript symbol * denotes complex conjugation. The introduced angles $\theta_{x}$, $\theta_{y}$ play a major role in the clear separation of all spectral components $\hat{A}$,$\hat{C}$ and ${\hat{C}}^{*}$ in the Fourier domain and must be sufficiently high to avoid any overlap. On the other hand, angles cannot be too high, in order to meet the Nyquist criterion.

Once the spectral components are separated, using a bandpass filter around the specific spatial frequencies, it is possible to filter out the desired spectral component $\hat{C}$ containing the interference phase φ. By filtering only the desired component, the filtered spectrum ${\hat{C}}_{F}$ (subscript *F* denotes filtered component) is no longer Hermitian, so its inverse Fourier transform will contain non-zero both real and imaginary parts, and hence the interference phase can be calculated as:

(10) $$\varphi \left(x,y\right)=\arctan\left(\frac{Im\left(C_{F}\left(x,y\right)\right)}{Re\left(C_{F}\left(x,y\right)\right)}\right).$$

The phase along with other quantities is stored in a computer’s memory as a matrix of numbers. Let us assume a measurement at a steady/reference state without the presence of fluid flow. Quantities measured in the reference state can be denoted using a subscript 0, e.g., $\varphi_{0}$ for the interference phase. Such a measurement carries information about interferometer optical aberrations, including aberrations introduced by, e.g., optical windows of the wind tunnel experiment. Other measurements performed with the presence of a phenomenon (denoted with subscript 1) can be related to the reference state measurement in order to suppress undesired optical aberrations. The phase change Δφ occurring in (1) can be calculated as:

(11) $$\Delta \varphi \left(x,y\right)=\arctan\left(\frac{Im\left(C_{F1}\left(x,y\right)C^{*}{}_{F0}\left(x,y\right)\right)}{Re\left(C_{F1}\left(x,y\right)C^{*}{}_{F0}\left(x,y\right)\right)}\right).$$

It is worth noting that spatial carrier interferometry could also be called off-axis image plane digital holographic interferometry. As phenomena in fluid flow do not lead to speckles, and there is also no need for the numerical propagation/focusing/reconstruction of wave fields typical for digital holography, we tend to call it interferometry.

## 3. Materials and Methods

**Figure 2:**
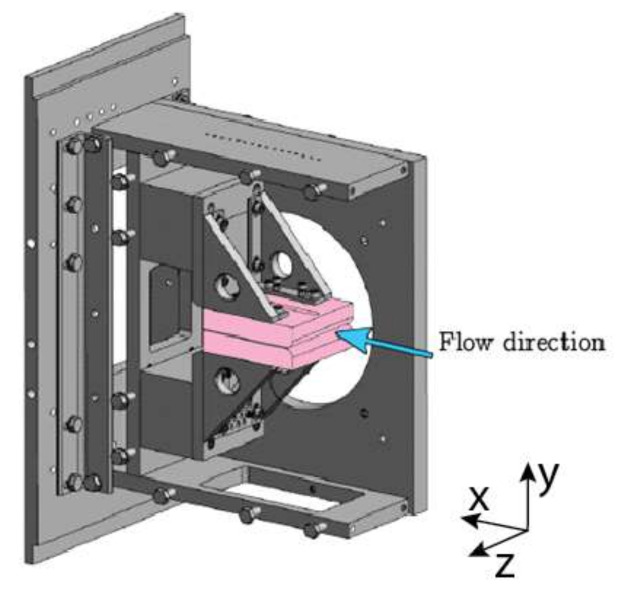
3D CAD assembly of the narrow channel facility.

Interferometry-based research of a fluid flow in a narrow channel including a jet flow at its outlet was carried out at the Laboratory of Internal Flows of the Institute of Thermomechanics of the Czech Academy of Sciences. The laboratory is equipped with a modular in-draft wind tunnel facility that enables low mass flow rates. The vacuum tank is connected to the test section through a pipe with a quick-acting valve and a nozzle that offers the possibility of changing the pressure inside the testing section from 0.01 MPa to values close to those of the ambient atmospheric pressure. The three-dimensional computer-aided design (3D CAD) assembly of the narrow channel is shown in Figure 2.

The experimental arrangement was designed in order to maintain a constant refractive index along the z-axis, i.e., the optical axis. Two parallel walls made of stainless steel constitute the flow channel. The test section has optical windows on the walls, allowing the passage of light beams for optical measurements. The channel height (along the y-axis) was set to 3 mm. The channel is 100 mm long (x-axis) and the width (z-axis) of the channel is L=100 mm to achieve a sufficient aspect ratio to eliminate secondary flow effects and to provide the sufficiently long optical path necessary for the adequate sensitivity of the interferometry. In order to obtain quantitative values of measurements, the ambient atmospheric pressure ($P_{in}$) as well as the outlet static pressure in the settling chamber of the wind tunnel ($P_{out}$) were measured.

**Figure 3:**
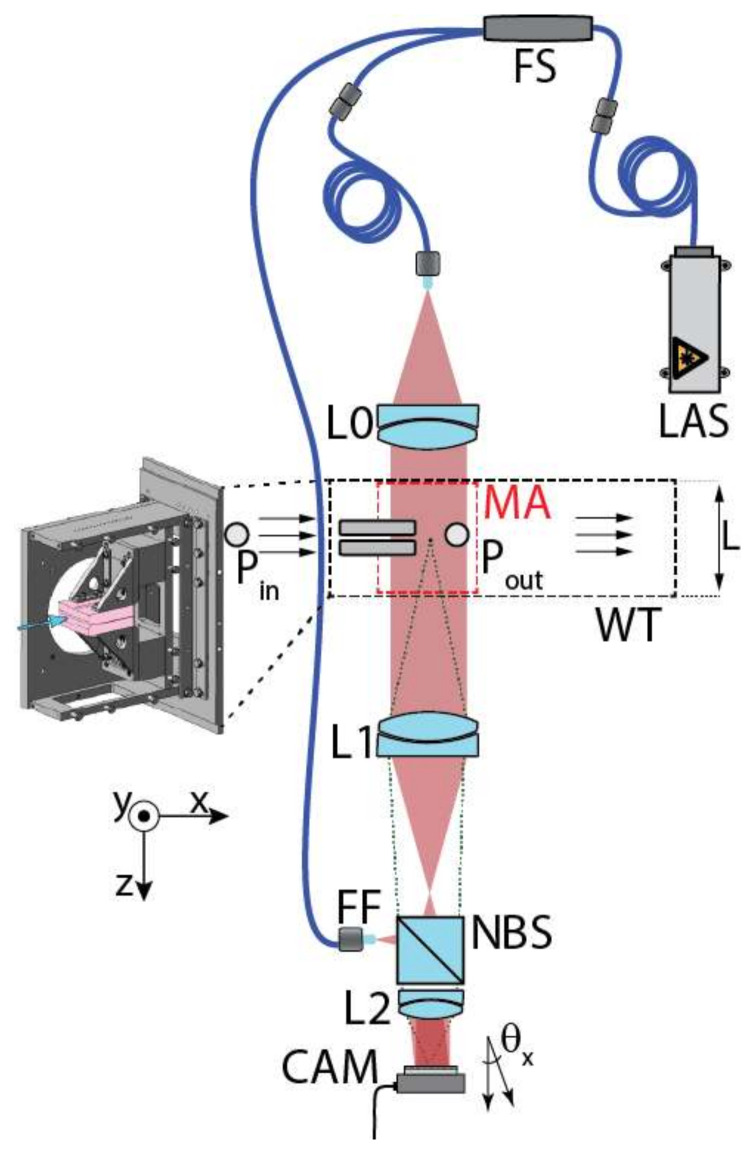
Scheme of the setup: LAS—laser, FS—fiber splitter, FF—fiber ferrule, L0 + L1 + L2—lens, MA—measured area, P0 + P1—pressure probes, WT—wind tunnel, NBS—beamsplitter, CAM—camera.

The experimental setup, consisting of the narrow channel experimental section and a Mach–Zehnder interferometer, is illustrated in Figure 3. A laser beam was generated by a pigtailed single-frequency distributed feedback laser (LAS) of wavelength λ=773 nm and fiber output power of 38 mW. The laser beam was split using a fibersplitter (FS) into the reference wave and the object wave. The reference wave was guided by an optical fiber to the non-polarizing beamsplitter (NBS), while the free-space object wave passed through a collimating imaging lens L0, which sent light through the measuring area (MA) within the wind tunnel (WT), before it passed through the lens L1. Lenses L1 and L2 acted as a beam expander with magnification of Mag≈0.6 as well as an imaging system. Both reference and object waves were recombined by the NBS and collimated by L2. The object wave impinged the digital camera sensor (CAM) normally, while the reference wave angles were $\theta_{x}=0.34{}^{\circ},\;\theta_{y}=0.30{}^{\circ},$ respectively. The angles of the reference wave were adjusted by a slight translation of the reference beam’s fiber ferrule (FF).

The intensity interference pattern (interferogram) of both superposed beams was captured by a Phototron FASTCAM Mini WX100 high-speed camera with a resolution of 2048×2048 pixels (10 μm×10 μm pixel size) and a frame rate of 1080 fps. The exposure time of 10 μs was set in order to avoid the blurring of the interference pattern while maintaining sufficient brightness (signal).

**Figure 4:**
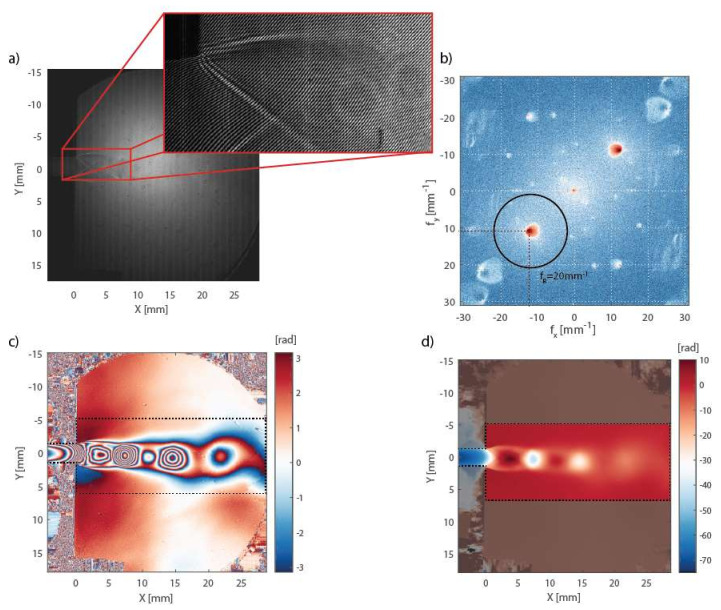
(**a**) Intensity image (interferogram) captured during measurements, with the zoomed part showing the interference pattern, (**b**) its Fourier spectrum, (**c**) the phase change map with region of interest (ROI) marked by the dashed line, and (**d**) unwrapped phase change map with ROI.

In the first step, a reference interferogram at a steady state ($P_{out}=P_{in}$) was captured and processed, and the filtered complex field $C_{F0}$ was stored in a computer. The same procedure was repeated after a change in the pressure inside the testing section ($P_{out}\neq P_{in}$), resulting in air flow. In order to obtain enough data for statistical analysis, a sequence of M=50 interferograms was captured, of which one is presented in Figure 4a. The amplitude Fourier spectrum of the interferogram in the logarithmic scale is shown in Figure 4b. The terms $\hat{A,}\;\hat{C,}\;{\hat{C}}^{*}$ are well separated by the spatial carrier frequencies $f_{x0}=\frac{k_{x0}}{2\pi }=\frac{sin\theta_{x}}{\lambda M}=12.3\;{\mathrm{mm}}^{-1}$ and $f_{y0}=10.8{\;\mathrm{mm}}^{-1},$ respectively. The black circle represents the filtering bandpass Hanning window, with bandwidth $f_{B}=20{\;\mathrm{mm}}^{-1}$, applied in order to retrieve the complex field $C_{Fm}$, where m=1,2…M is an integer denoting the frame number and M is the number of captured interferograms. Such bandwidth results in a spatial (lateral) resolution of about 50 μm. It is important to note that the applied filtering must remain unchanged from that in the reference interferogram processing. The optical phase change computed by (11) is wrapped within the $\left[-\pi ,\;\pi \right]\;radians$ interval due to the harmonic nature of the light wave—see Figure 4c. Therefore, a spatial unwrapping was applied with the starting point in the position of the Pitot probe, i.e., far from the channel without the influence of the flow, obtaining a sequence of unwrapped phase fields $\Delta \varphi_{m}$—see Figure 4d. In order to suppress the random time fluctuations of the flow, the mean of the unwrapped phase was computed:

(12) $$\Delta \varphi =\frac{\sum_{m=1}^{M}\Delta \varphi_{m}}{M}$$

The mean unwrapped phase change distribution Δφ was used to calculate the density distribution as described in Section 2.1.

In addition to the interferometric measurements, computational fluid dynamics (CFD) simulation software was used to cross-check both the measurement and the simulation. The numerical computations were performed using Ansys Fluent 2021R1 commercial code. This software package uses the finite volume method to solve the governing Navier–Stokes equations for compressible fluid flow. The problem was simulated in two dimensions (2D) using the Reynolds-averaged Navier–Stokes equations (RANS) approach to close the system of equations. The flow of air as an ideal gas was simulated as fully turbulent using the two-equation k-ω shear stress transport (SST) turbulence model. The computational mesh consisted of approximately 150,000 cells. The mesh sensitivity analysis was performed using two finer meshes, yielding a relative error of the mass flow rate of 0.3% for the coarse mesh, which is very acceptable. The computational domain was assumed to be symmetrical around the channel axis with the no-slip boundary condition at the channel walls. The static pressure according to the desired pressure drop across the channel was prescribed as the outlet boundary condition. The following boundary conditions were used at the inlet: (a) constant total temperature $T_{0}=292.67\;K,$ (b) uniform total pressure distribution $p_{0}=98.05\;\mathrm{kPa},$ (c) constant turbulence intensity Tu=1.2% and (d) constant eddy viscosity ratio $\frac{\mu_{t}}{\mu }=10$.

## 4. Results and Discussion

The results of the interferometric measurement of air flowing out of the narrow channel, and their comparison to the results of CFD simulation, are presented in this section.

The fluid flow in the narrow channel was investigated in five different regimes defined by different pressure gradients through the channel. The magnitude of the pressure gradient was controlled by a valve that determined the outlet pressure in the settling chamber downstream of the narrow channel. Both inlet and outlet pressure were measured, yielding the pressure ratio $\eta =\frac{P_{out}}{P_{in}}$. The inlet pressure (the ambient atmospheric pressure) was constant during all measurements: $P_{in}=98.050\;\mathrm{kPa}$. Parameters under different regimes are summarized in Table 1.

Other constants considered for further flow analysis and CFD simulations are the ambient temperature $T_{0}=295.9\;K$, the Gladstone–Dale constant $K=0.000225\;m^{3}{\mathrm{kg}}^{-1}$, and the specific gas constant of dry air $R=287.1\;J/\left(\mathrm{kg}\cdot K\right)$.

**Figure 5:**
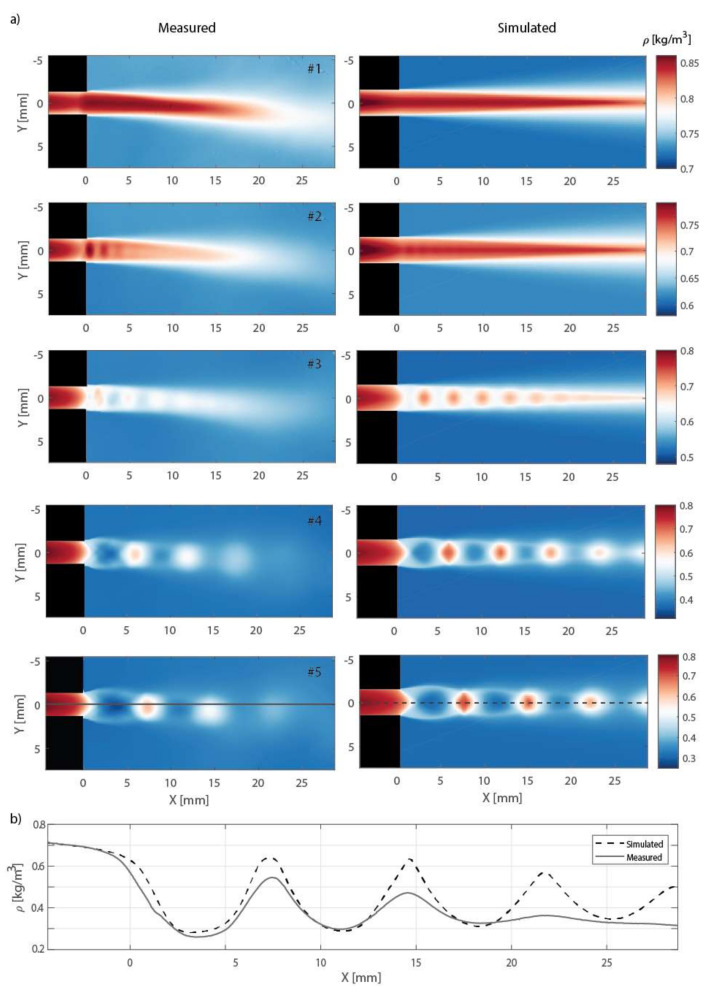
(**a**) Density distributions for different regimes—comparison of measured data (**left**) and the CFD simulations (**right**); (**b**) density values along the profile denoted in (**a**) for regime #5.

The density distributions computed using (5), together with the results of the CFD simulation, are presented in Figure 5. It can be seen that the interferometric measurements and CFD simulations are in a very good agreement. Small discrepancies can be attributed to minor asymmetries in the geometry of the experimental setup. The boundary conditions of the CFD simulation could be modified in order to derive an even better agreement; however, this is not this paper’s objective.

**Figure 6:**
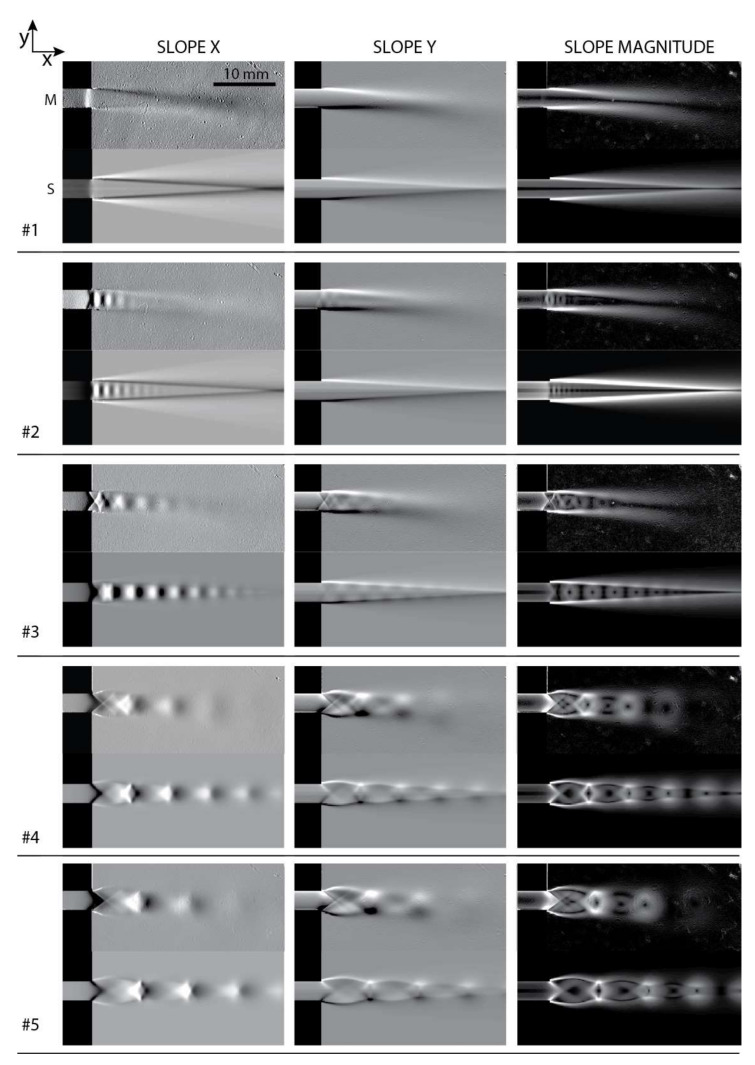
Slope maps of measured (top—M) and simulated (bottom—S) density distributions in different flow regimes.

The measured distributions in Figure 5 have significantly dynamic ranges of values. Therefore, some faint details of the flow might be difficult to observe. In order to visualize the high-frequency spatial variations in the flow, the slopes of the measured density distribution in the horizontal $\left(x\right)$ and vertical $\left(y\right)$ directions $\partial \rho_{M}\left(x,y\right)/\partial x$,$\;\partial \rho_{M}\left(x,y\right)/\partial y$ were calculated, and these, together with the magnitude of the slope $\sqrt{{\left(\partial \rho_{M}/\partial x\right)}^{2}+{\left(\partial \rho_{M}/\partial y\right)}^{2}}$, are shown in Figure 6. Such results could be directly compared to the Schlieren imaging techniques, which are sensitive to the ray deflections caused by refractive index gradients $\partial n\left(x,y\right)/\partial x$ and $\partial n\left(x,y\right)/\partial y$, respectively.

Five different regimes with a gradually decreasing ratio η were measured. In the first regime, #1 (η=0.560), the supercritical pressure ratio across the channel results in the subsonic discharge of air into the settling chamber. Thus, a subsonic exit jet can be seen in Figure 6. There is also visible compression just before the exit of the channel. This indicates that the regime is close to aerodynamic choking, and is approaching supersonic velocity within the channel.

In regime #2 (η=0.481), the exit jet is supersonic with a shock wave present. Thus, the flow is already aerodynamically choked. In this regime, the flow is most likely overexpanded (i.e., the pressure at the exit cross-section of the channel is lower than the outlet pressure in the settling chamber), since two oblique shocks are formed at the exit of the channel first.

In the supersonic regime #3, as the ratio further decreases (η=0.400), the jet flow’s inner pattern starts to take an “X” or a “diamond” shape, which is characteristic of a moderately underexpanded jet flow. Here, expansion waves form at the edge of the channel, causing the flow to turn outward in a plume, creating a characteristic underexpanded jet flow pattern. Expansion waves “reflect” from the jet’s constant pressure boundary as compression waves, which eventually form oblique shock waves. A periodical series of compression and expansion waves appears, until the jet is dissipated and static pressure within the jet flow reaches equilibrium with the surrounding pressure (backpressure).

In the supersonic regimes #4 (η=0.287) and #5 (η=0.243), the flow first expands through expansion waves, before turning into oblique shocks and Mach disks. Such highly underexpanded flow regimes exhibit a “barrel-shaped” pattern that differs in the shapes and lengths of the shock cells with different η. The flow patterns repeat until mixing dissipates the flow.

The typical resolution of the optical phase measured by interferometry is better than 1/100 of 2π, leading to a refractive index resolution of about $4\times 10^{-7}$ in this particular case. However, interferometric measurements in a wind tunnel are burdened with errors. The first group of errors is directly due to interferometric sensing—the measurement of the optical phase. When retrieving Δn from (2), there is an error in laser wavelength $u_{\lambda }$, thickness $u_{L}$, and phase noise $u_{\Delta \varphi }$. Let us define the refractive index combined error as:

(13) $$u_{\Delta n}=\left|\frac{\partial \Delta n}{\partial \Delta \varphi }\right|u_{\Delta \varphi }+\left|\frac{\partial \Delta n}{\partial \lambda }\right|u_{\lambda }+\left|\frac{\partial \Delta n}{\partial L}\right|u_{L}$$

The phase noise for our measurement is estimated to be $u_{\Delta \varphi }=0.07\;\mathrm{rad}$. This value was derived from experimental data as the standard deviation of an unwrapped phase map (in different regions) after low-order polynomial subtraction (i.e., high-spatial frequency components were considered as noise). The wavelength of the used laser source depends on the temperature and current of the laser diode. The temperature and the current are measured/controlled; however, there is not a specific relation between these and the output wavelength, thus there is a wavelength error, which can be estimated to be $u_{\lambda }=0.5\;\mathrm{nm}$. The error of thickness, $u_{L}=0.1\;\mathrm{mm}$, comprises, on one hand, the limited accuracy of channel manufacturing, and on the other hand the fact that rays are deflected due to refractive index non-homogeneities. Such ray deflection is not considered in (2). Moreover, in locations with a large refractive index gradient, the ray deflection misplaces information geometrically (up to 200 μm), leading to so-called mapping errors. Such distortion is below ten pixels in the areas with the largest gradients, otherwise it is negligible. By substituting in (13), the maximal error is $u_{\Delta n}\approx 1\times 10^{-6}$ and the relative maximal error results in $100\frac{u_{\Delta n}}{\Delta n}\approx 2\%$.

Assuming the refractive index error $u_{\Delta n}$, along with the 2% relative error of the pressure/temperature sensors and 1% error of the Gladstone–Dale constant in (5), the density error yields $u_{\rho_{M}}\approx 0.015\;\mathrm{kg}/m^{3}$, or relatively $100\frac{u_{\rho_{M}}}{\rho_{M}}\approx 2.6\%$.

**Figure 7:**
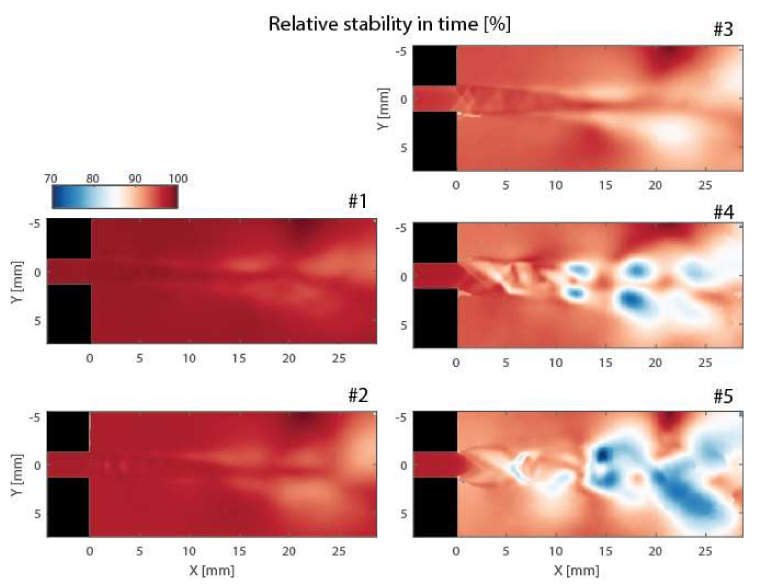
The time stability of the flow under different regimes.

Another source of error is usually related to the repeatability of measurements. However, the repeatability of measurements in this particular case can only be determined under a steady state (without flow) due to the unstable behavior of the flow. Steady state repeatability has a negligible effect when compared to the stability of the flow. Therefore, time stability was examined. As aforementioned, we captured 50 frames during one measurement sequence, taking 50 ms, and used averaged phase maps Δφ for further processing—see (12). However, the standard deviation,

(14) $$\sigma \left(x,y\right)=\sqrt{\frac{\sum_{m=1}^{M}{\left(\Delta \varphi_{m}\left(x,y\right)-\Delta \varphi \left(x,y\right)\right)}^{2}}{M-1}},$$

for each pixel of $\left(x,y\right)$ coordinates can also be computed from the data set. The standard deviation can be used to calculate a time stability map $\tau \left(x,y\right)==1-3\sigma \left(x,y\right)$ that (assuming the normal probability distribution) equals one in pixels of completely stable flow, while it approaches zero at locations of random signal. The time stability maps of flow in different flow regimes are shown in Figure 7. It is clear that in the narrow channel, the flow is very stable, but becomes much less stable in shear layers and regions of flow dissipation. This effect is more apparent at high flow velocities with a more turbulent character.

## 5. Conclusions

This paper presents a very effective technique for sensing and investigating compressible fluid flow. The developed interferometric technique is very sensitive and accurate, yet easy to use, even under pretty harsh environmental conditions (vibrations, flow instability, unclean room) typical for aerodynamic labs. The interferometer employs a high-speed camera, fiber optics, and readily available “of-the-shelf” optics and optomechanics. The interferometer consists of an illumination and a sensing unit, each weighing less than 5 kg. An optical fiber delivers the reference wave from the illumination to the sensing unit, while the object wave emitted from the illumination unit propagates through a measured volume into the sensing unit. Both waves are superimposed, and an interference pattern is captured by a high-speed camera. The construction of the interferometer together with the fiber optics ensures the high compactness and portability of the system. Moreover, single-shot quantitative data processing based on introducing a spatial carrier frequency and processing in the Fourier domain allows for almost real-time quantitative processing, which is also suitable for very fast-evolving phenomena.

The interferometer was successfully applied for the study of a fluid flow in a narrow channel including a free jet at the channel exit. The experimental section was built in a wind tunnel, allowing for a high pressure difference and thus high flow velocity through the channel. The flow was investigated under five different regimes, ranging from subsonic to supersonic flow with a highly underexpanded jet. The density distributions were quantitatively analyzed with a spatial resolution of about 50μm. The results of the measurement were able to reveal important features of the flow pattern, such as shock waves and slip lines.

The measurement results were compared with CFD simulations, showing good agreement in terms of the flow pattern similarity (e.g., size of the shock cells) as well as the absolute values of density. An error analysis revealed the error range in units of percent. It can be concluded that the method constitutes a promising tool for studying highly dynamic events, even during transonic and supersonic blade fluttering.

## Figures and Tables

**Table 1:** Regimes and the corresponding pressure and pressure ratio values.

Regime	#1	#2	#3	#4	#5
P_out_ (kPa)	54.923	47.140	39.236	28.114	23.871
η [1]	0.560	0.481	0.400	0.287	0.243

## Data Availability

The data presented in this study are available on request from the corresponding author.
